# Case of Cerebral Extravasation [Extrusion?] in a New-born Infant, Followed by Spontaneous Recovery

**Published:** 1846-04

**Authors:** Hattersly P. Worthington

**Affiliations:** Elkridge Landing, Maryland


*Case of Cerebral Extravasation* [*Extrusion?*] *in a New-born Infant, followed by Spontaneous Recovery.* By HATTERSLY P. WORTHINGTON, M. D., of Elkridge Landing, Maryland.

January 27th, 1846, at 1 o'clock, P. M., I was called to attend Mrs. L——, æt. 20, in labour with her first child. She had had moderate labour pains since 11 o'clock of the previous night. Upon examination, I found the head presenting in the first position, (Velpeau;) the *os tincæ* slighly dilated; the pelvis below the medium size, but without any deformity of shape. The uterine efforts became more decided, were frequent, and of sufficient power to cause a steady advance of the head. Although the labour was tedious, I saw no necessity for artificial assistance, but left the case to nature, and at 3 o'clock the next morning the child—a fine male, weighing nine pounds—was delivered. The head appeared more than usually elongated, with a soft tumour, of the size and shape of half of a hen's egg, over the lamdoid suture, to the right of the posterior fontanelle. As the opposite side of the head assumed its natural shape, this tumour continued to occupy its situation, pressing out through the suture, and separating the bones from each other. At the expiration of two weeks it remained without change, feeling soft, without any perceptible fluctuation.

My esteemed friend, Dr. Robert E. Dorsey, now saw it with me, and we were induced to believe there was an arrest of bony formation on this part of the skull. We determined to do nothing for the present—the child enjoying good health—but to await the occurrence of events requiring interference.

On the 16th of February I observed an evident decrease in the size of the tumour; the following day this was more perceptible, and on the 18th it had entirely disappeared, and the sutures had approximated as nearly as upon the opposite side. The child continues to thrive, having apparently suffered but little inconvenience from this rather unusual freak of nature to facilitate its passage into the world. The labour was attended with consequences to the mother, the relation of which I shall reserve as the subject of a future paper.

*February 23d*, 1846.

